# Development of the Korean Medicine Core Outcome Set for Primary Dysmenorrhea (COS-PD-KM) for Herbal Medicine Treatment of Primary Dysmenorrhea in Primary Clinics

**DOI:** 10.3390/ijerph192215321

**Published:** 2022-11-19

**Authors:** Pyung-Wha Kim, Sungha Kim, Dong-Il Kim, Jiyun Cha, He-Sol Lee, Mi Mi Ko, Soobin Jang, Changsop Yang, Myeong Soo Lee

**Affiliations:** 1 R&D Strategy Division, Korea Institute of Oriental Medicine, Daejeon 34054, Republic of Korea; 2 KM Science Research Division, Korea Institute of Oriental Medicine, Daejeon 34054, Republic of Korea; 3 Department of Obstetrics & Gynecology, College of Korean Medicine, Dongguk University, Goyang 10326, Republic of Korea; 4 Digital Health Research Division, Korea Institute of Oriental Medicine, Daejeon 34054, Republic of Korea; 5 Department of Preventive Medicine, College of Korean Medicine, Daegu Haany University, Gyeongsan 38610, Republic of Korea

**Keywords:** core outcome set, primary dysmenorrhea, primary care, herbal medicine, Korean medicine

## Abstract

The aim of this study was to develop a Korean medicine (KM) core outcome set (COS) for primary dysmenorrhea to evaluate the effectiveness of herbal medicine (HM) in treating primary dysmenorrhea in patients visiting KM primary clinics. Previously reported outcomes were identified through a literature review to define outcomes and effect modifiers (EMs) for the questionnaire. Experts were invited to conduct modified Delphi consensus exercises, and primary care clinicians were invited to conduct Delphi consensus exercises to evaluate suitability and feasibility. Finally, an additional round of a modified Delphi exercise was conducted with experts to obtain a final agreement on the COS. Seventeen outcomes and 15 EMs were included from a literature review, and one effect modifier was suggested by the experts (Phase 1). In Phase 2, after the modified Delphi consensus exercises by experts, 10 outcomes and 11 EMs were included in the COS. The clinicians all agreed on the feasibility of COS (Phase 3). Finally, 10 outcomes and 6 EMs were included in the COS-PD-KM after the final modified Delphi consensus exercise (Phase 4). The effectiveness of HM used in primary clinics could be evaluated with this COS in patients with primary dysmenorrhea. Further studies that involve more relevant stakeholder groups, such as patient representatives and gynecological experts, are needed.

## 1. Introduction

Primary dysmenorrhea (PD) is defined as painful menstrual cramps without clear pathological pelvic disease [1]. This debilitating condition has a negative effect on women’s daily activities, leading to a lower health-related quality of life [2,3]. Nonsteroidal anti-inflammatory drugs (NSAIDs) and oral contraceptives (OCPs) are readily accessible first-line treatments for dysmenorrhea [4,5]. These drugs control pain by inhibiting prostaglandins (PGs). However, the long-term use of NSAIDs is strongly associated with side effects, such as gastrointestinal discomfort, vomiting, headache, and negative effects on renal function [4].

Herbal medicine (HM) is widely used for women’s healthcare [6,7,8,9,10,11]. Korean medicine (KM) is the conventional medicine used in South Korea, and most treatments are covered by the national health insurance system. The main treatments in KM consist of acupuncture, HM and cupping. Although HM is not covered by health insurance in South Korea, in a previous survey of the practice habits of KM physicians, HM was the most commonly used treatment for patients with PD [12]. HM, such as the Guizhi Fuling Wan and Shaofu Zhuyu decoction, was shown to have analgesic effects by reducing uterine contractions and inhibiting PG synthesis by reducing COX2 or nitric oxide (NO) in female mice with oxytocin-induced dysmenorrhea [13,14,15]. Previous systematic reviews have reported the promising effects of HM on improving the symptoms of dysmenorrhea [9,10,11,16,17]. However, the evidence is suggestive, and large-scale clinical trials are needed to investigate the efficacy of HM for PD. Because most Korean medical facilities are primary clinics, clinical trials in KM clinics are inevitably insufficient in terms of personnel and equipment, resulting in low-quality studies that examine inappropriate outcomes. In 2020, the Korean Government launched a pilot project to cover HM in patients with PD in primary clinics with national insurance. The listed HM was selected based on the evidence in the “Dysmenorrhea Korean Medicine Clinical Practice Guideline” [12] and is described in detail in Supplementary Table S1. Health insurance now covers the cost of 10 days of HM treatment that meets government diagnostic and quality standards. However, this project lacks a standard outcome model to evaluate the effect of HM on PD. Therefore, an outcome model suitable for a primary care setting that evaluates the therapeutic effect of HM using consistent and appropriate evaluation variables must be developed. The aim of the study was to develop a KM Core Outcome Set (COS) for PD (COS-PD-KM) to evaluate the effectiveness of HM as a treatment for primary dysmenorrhea in patients attending KM primary clinics.

## 2. Methods

A COS was developed based on the Recommendations of the COS Standards for Development (COS-STAD). Additionally, effect modifiers (EMs), as important factors influencing outcomes, were extracted to allow clinicians to investigate them during history taking. We followed the development process of a COS reported by the COMET initiative and COS standard guidelines [18]. It consists of four phases based on the COS for stroke sequelae previously published by our team [19]. First, we established a project management group (PMG) and created and refined a list of outcomes based on a literature review. Second, experts conducted a modified Delphi consensus process. Third, primary clinicians conducted a modified Delphi consensus exercise. Finally, experts finalized the COS lists through the final Delphi consensus exercise.

### 2.1. Phase 1: Establishing a Study Advisory Group and Generating a Comprehensive List of Outcomes and EMs

We established a PMG consisting of five researchers from the Korea Institute of Oriental Medicine to conduct a literature search, extract outcome/effect modifiers, remove duplicate results, and so on. The scope of COS was clarified, and an overview of systematic reviews (SRs) and meta-analyses was conducted. The process of SR is described in detail in the Supplementary Materials (Tables S2 and S3; Figure S1).

SRs and meta-analyses related to HM trials in women with PD were included in this study, and the inclusion criteria and the outcomes of the original RCTs were independently double-checked. The numbers of RCTs, analyzed participants, interventions, comparators, and outcomes of the included studies were extracted. Disagreements were resolved by discussion.

Additionally, previously published books, such as “Dysmenorrhea Korean Medicine Clinical Practice Guideline”, “Oriental Obstetrics & Gynecology”, and articles on risk factors for dysmenorrhea were reviewed to extract the recommended outcomes and EMs for PD.

Duplicate studies were removed. The PMG reviewed the list of outcomes and EMs in accordance with the rationale of this COS to produce the final list for Phase 2.

### 2.2. Phase 2: Consensus in Experts

We recruited experts in KM gynecology, who are professors of KM at a university or clinicians in a KM hospital that specializes in KM gynecology, to participate in the Delphi survey. The Society of Korean Medicine Obstetrics and Gynecology (SKMOG) recommended six experts for the panel by request from the PMG. Only five finally agreed to participate in the Delphi process after being informed of the rationale of the COS.

We distributed questionnaires with group feedback and meetings to achieve a consensus. In Round 1, the outcomes and EMs screened in Phase 1 were sent to the panel to rate the importance of each item. We used a 4-point numerical scale to avoid choosing the middle value. Additionally, panels were able to suggest outcomes or EMs that were not on the list. The score distribution was calculated. In Rounds 2 and 3, the panels discussed all outcomes and EMs, including those proposed in Round 1, taking into account the panel responses to determine those appropriate for the scope of COS. Discussion continued until a consensus was reached.

### 2.3. Phase 3: Consensus in Primary Clinicians

Primary clinicians representing some of the key stakeholders reviewed the feasibility of the derived outcomes and EMs by completing one round of a Delphi process.

At the request of the PMG, the Korean Medicine Specialists Association recommended panels for KM primary clinicians with at least five years of clinical experience. Finally, eleven specialists participated in the panel, two of whom specialized in KM gynecology.

The derived EMs and outcomes were reviewed and evaluated in terms of survey feasibility in KM primary clinics. The questionnaire consisted of two sections: (1) EMs for menstrual history and related gynecologic history and (2) outcome measures (numeric rating scale (NRS) for pain, pain duration, analgesic consumption, EQ -5D-5 L and EQ-VAS, and satisfaction measures). A 9-point scale was used.

Items such as laboratory test results (AST, ALT, BUN, and Cr levels) and adverse events were excluded from Phase 3 because the PMG discussed them and concluded that they were essential COS items for investigating the safety and effectiveness of HMs.

### 2.4. Phase 4: Final Consensus in Experts

We conducted the final modified Delphi exercise reflecting the results of Phase 3 and the opinions of clinicians to increase the feasibility of evaluating the outcomes in the clinical field through the final consensus of experts. Key outcomes and EMs were selected based on the Delphi results.

The experts who participated in Phase 2 were asked to participate in Phase 4 again. We explained the results and opinions from Phase 3 to the experts by e-mail and asked whether they would participate in the final Delphi round for the final determination of the COS. All participants agreed to participate in the final Delphi survey.

The experts reviewed the results and qualitative opinions of Phase 3 and answered the Delphi questionnaire. Based on the results of Phase 3, the questionnaire was composed of the following sections: (1) EMs for menstrual history and related gynecological history (which were selected in the previous phase) and (2) outcome measures (NRS for pain, pain duration, and consumption of painkillers) to evaluate outcomes. Responses to the questionnaire were rated on a 4-point scale.

Outcomes such as quality-of-life evaluation (EQ-5D-5 L and EQ-VAS) and satisfaction measures were excluded from Phase 4 because the PMG discussed them and concluded that they were essential COS items to investigate the safety and effectiveness of HMs.

### 2.5. Data Analysis

#### 2.5.1. Phase 2: Consensus among Experts

In the modified Delphi consensus exercise with experts, unanimity was regarded a priori in the criteria for consensus. A unanimous decision was reached as either “consensus in” or “consensus out”.

#### 2.5.2. Phase 3: Consensus among Primary Clinicians

Similar to the COS previously published by our team for stroke outcomes [19], we used the content validity ratio (CVR) and the degree of agreement and convergence. The critical CVR value, degree of agreement, and convergence were ≥0.636, ≥0.75, and ≤0.5, respectively, for 11 Delphi panelists [20,21].

#### 2.5.3. Phase 4: Final Consensus among Experts

In Phase 4, the five experts from Phase 2 participated again in the final modified Delphi survey by completing a written questionnaire. The results of Phase 4 were analyzed using degrees of consensus and convergence, and the calculation of these two outcomes was the same as in Phase 3.

### 2.6. Ethics and Consent

The Institutional Review Board of the Korea Institute of Oriental Medicine, Daejeon, Republic of Korea, granted an ethical approval exemption (IRB approval no. I-2203/003-002). All participants gave written informed consent to participate in the study.

## 3. Results

### 3.1. Phase 1: Generating and Refining a Comprehensive List of Outcomes and EMs

The literature review identified 130 studies in the English and Korean databases. Twenty-five duplicate studies and 92 studies that did not meet the inclusion criteria were excluded after screening the title and abstract. After assessing the full text for admissibility, three additional studies were excluded, and finally, ten SR studies were included in the literature review. A total of 10 SRs were analyzed (Supplementary Tables S2 and S3).

Detailed information, including outcomes, was extracted from the 10 SRs. In the PMG review, outcomes for the response (or clinical/total effective rate) and recurrence rate, where the criteria for symptom improvement differed by individual investigators, were excluded. Sixteen outcomes were extracted from the literature review (Step 1). In the next step (Step 2), we extracted 11 additional outcomes and 14 EMs by reviewing relevant literature, such as “Dysmenorrhea Korean Medicine Clinical Practice Guide-line”, “Oriental Obstetrics & Gynecology”, and articles on EMs (or risk factors) of dysmenorrhea. Finally, the PMG reviewed Steps 1 and 2 and discussed the “in/out” articles according to the rationale for this COS to create the final list for Phase 2. After screening and removing duplicate entries in Phase 1, 17 outcomes and 15 EMs were included in the Phase 2 consensus exercise (Table 1).

### 3.2. Phase 2: Consensus among Experts

**Figure 1:**
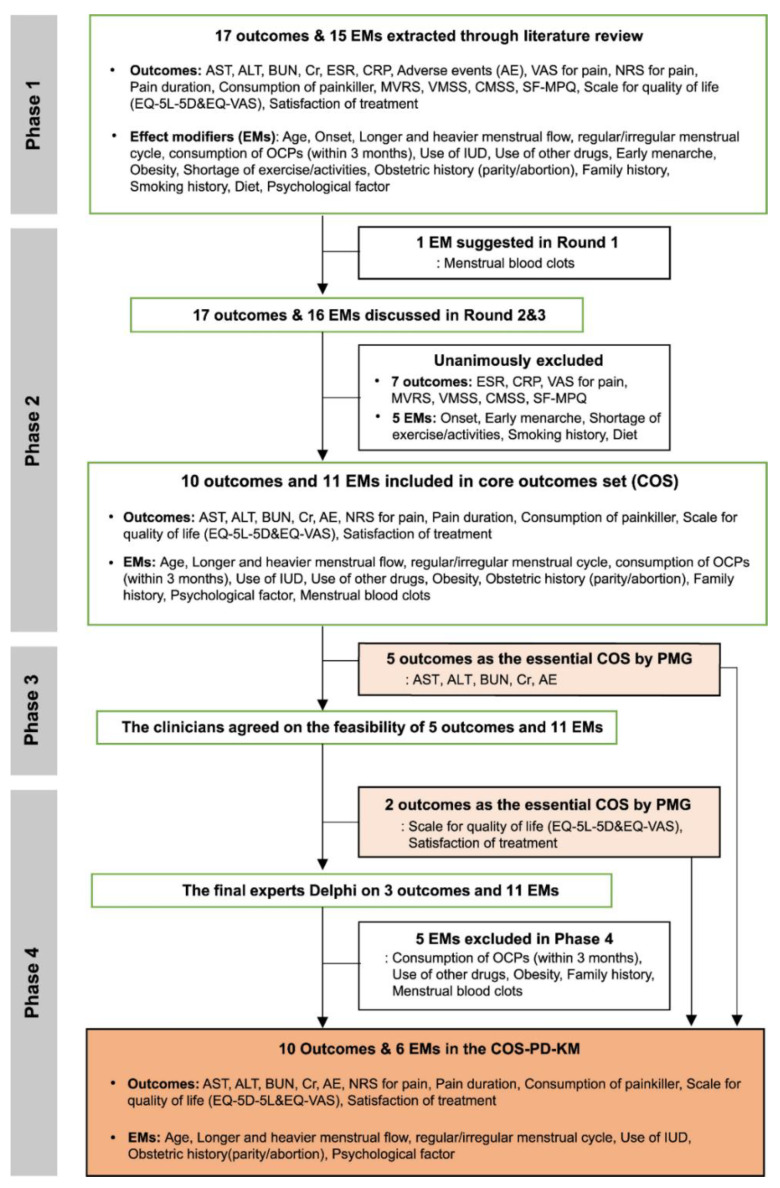
Overview of the development of the Korean Medicine Core Outcome Set for Primary Dysmenorrhea (COS-PD-KM). The expert panels reached a consensus through three rounds of Phase 2. AE: adverse events; AST: aspartate transaminase; ALT: alanine transaminase; BMI: body mass index; BUN: blood urea nitrogen; CMSS: COX Menstrual Symptom Scale; Cr: creatinine; CRP: C-reactive protein; ESR: erythrocyte sedimentation rate; EMs: effect modifiers; EQ-5 L-5D: EuroQol-5 Dimensions-5 Level; EQ-VAS: EuroQol visual analog scale; IUD: intrauterine devices; MVRS: Multidimensional Verbal Rating Scale; OCP: drug interaction oral contraceptive pill; SF-MPQ: Short-form of McGill pain questionnaire; VAS: Visual analog scale; VMSS: Verbal Multidimensional Scoring System.

Five experts in KM gynecology with ≥ 8 years of clinical experience participated in the Delphi exercise from November to December 2020. One effect modifier, menstrual blood clots, was also included after suggestions from the experts (Figure 1). In round 2, 10 outcomes were unanimously determined to be “consensus in”, while 11 EMs were determined to be “consensus in.” Regarding psychological factors, a consensus was reached that the measurement of the stress level encompasses anxiety and depression, and the experts agreed to measure the intensity of stress. Additionally, the experts suggested methods to evaluate the stress intensity and amount of menstrual blood, namely, by using the NRS and categorizing items, respectively. Finally, 10 outcomes and 11 EMs met the inclusion criteria for the consensus of the COS (Figure 1).

### 3.3. Phase 3: Consensus among Primary Clinicians

Because the CVR of all items, the primary outcome in this Delphi process, exceeded the critical value, the PMG concluded that all items were appropriate for survey in KM primary clinics. The NRS scores for pain and treatment satisfaction for the outcomes showed a sufficient level of consensus and convergence. All EMs exceeded the critical value for the level of agreement (Table 2).

### 3.4. Phase 4: Final Consensus in Experts

As a result of Phase 4, the three major outcomes (NRS for pain, pain duration for one menstrual cycle, and consumption of pain killers for one menstrual cycle) matched the degree of consensus and degree of convergence. Of the 11 modifiers, only 6 passed the standard and were finally selected (Table 3).

**Figure 2:**
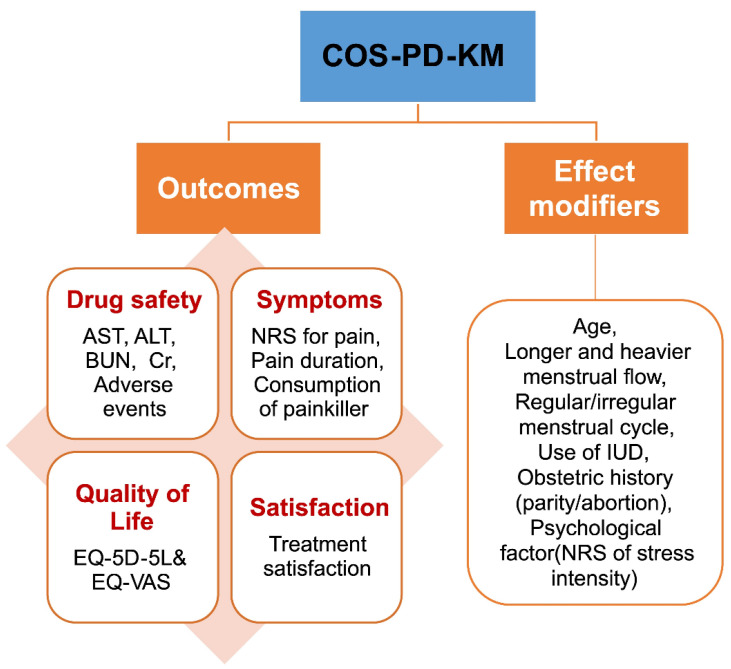
Conclusion of the Korean Medicine Core Outcome Set for Primary Dysmenorrhea (COS-PD-KM) development. Ten outcomes (categorized into drug safety, symptoms, quality of life, and satisfaction) and 6 modifiers were included in the COS-PD-KM. AST: aspartate transaminase; ALT: alanine transaminase; BUN: blood urea nitrogen; Cr: creatinine; EQ-5D-5 L: EuroQol-5 Dimensions-5 Level; EQ-VAS: EuroQol visual analog scale; IUD: intrauterine devices; NRS: numeric rating scale.

In summary, 10 outcomes and six EMs were included in the final COS together with the outcomes that the PMG, concluded to be the essential COS in the previous phases (Figure 1). The final outcomes include: (1) NRS for pain, (2) pain duration for one menstrual cycle, (3) consumption of pain killers for one menstrual cycle, (4) AST level, (5) ALT level, (6) BUN level, (7) Cr level, (8) adverse events, (9) quality of life (EQ-5D-5 L and EQ-VAS), and (10) satisfaction with treatment. The final EMs were (1) age, (2) longer and heavier menstrual flow, (3) regular/irregular menstrual cycle, (4) use of an IUD, (5) obstetric history (parity/abortion), and (6) psychological factor (NRS for stress intensity) (Figure 2).

## 4. Discussion

### 4.1. Summary of the Main Results

We developed a COS for HM in patients with PD in KM primary clinics. Based on the COMET initiative guidelines, a literature review was conducted by the PMG (Phase 1), and a modified Delphi exercise was conducted by gynecologic experts (Phase 2). Delphi surveys were then conducted by Korean Medicine Doctors (KMDs) (Phase 3) to assess the feasibility of COS. Finally, in Phase 4, the last modified Delphi survey of gynecologic experts led to the final results and EMs. The final results for COS-PD-KM are shown in Figure 2.

### 4.2. Considerations for COS Development

In the COS development process from Phase 1 to Phase 4, we conducted research focusing on the disease characteristics of PD and its feasible application in the primary medical field. Menstrual pain is the most common gynecological disorder experienced by 50% of reproductive-aged women, and its symptoms are repetitive and depend on the menstrual cycle stage [1]. PD is usually controlled by treatment with NSAIDs, OCPs, and HM within 4–12 weeks, but it is likely to recur due to general health conditions and psychological factors such as stress. Additionally, pain may be alleviated after pregnancy and childbirth. Hence, all discussions by the PMG and experts were based on the common opinion that obstetric and gynecological investigations and various physical and psychological factors must be considered during COS development.

The COS was considered for use in KM clinics, which lack the employees and equipment to conduct research, as in the case of the previously developed KM-COS for stroke sequelae [19]. Thus, we prioritized the feasibility of using the COS in KM clinics. A previous study conducted in KM clinics used simple outcomes, with only a small number of included outcomes to avoid interference with routine practice [22,23]. Simple but accurate outcomes with less disruption to clinical practice are recommended for the COS.

### 4.3. Outcomes Included in the COS and Their Implications

EMs are recommended for a further analysis of the measurement outcomes according to the characteristics of patients and should be investigated during history taking by clinicians. Information on age, longer and heavier menstrual flow, regular/irregular menstrual cycle, use of IUDs, obstetric history (parity/abortion), and psychological factors (NRS of stress intensity) was included as EMs in this project. Additionally, we decided to record the last menstrual period (LMP), as it is the basic information needed for treating PD.

Psychological factors (NRS of stress intensity) are included in the COS since a significant relationship between some mental health components, such as stress and PD, has been reported [24]. Despite its high prevalence, PD is often poorly treated and even disregarded by health professionals, pain researchers, and women themselves, who accept it as a normal part of the menstrual cycle [25]. Among patients with PD, painful menstruation in the absence of pelvic pathology is the chief complaint. Since patients can readily take NSAIDs or painkillers, measurements of the VAS for pain and medication dosages are important [26]. Dysmenorrhea pain has an immediate effect on the quality of life of women, and this effect lasts for a few days per month [25]. Hence, an estimation of the quality of life of patients with PD is important. Considering the association between menstrual cycles and pain, outcomes should be regularly measured in all patients, according to their personal cycles. Thus, all experts agreed on setting the outcome measurement time after patients took HM to ‘the end of the first menstrual cycle after HM medication’, but they also expected difficulty in achieving this outcome due to the limited workforce and time of the primary KM clinics.

### 4.4. Implications for the Development Process

The expert panel comprised KMDs, experts who best understood the purpose and characteristics of this study. Since the goal of this study was to develop a COS suitable for KM primary care, knowledge of PD and a thorough understanding of the characteristics of KM are needed. In addition, with a high proportion of physicians receiving training in Western medicine (WM) in KM schools, KMDs have taken a more active role in joint KM and WM care, as previous studies have shown [27,28].

This COS was specifically developed for use in primary clinics HM. Because our primary concern was feasibility in primary clinics, simple and easy outcomes and EMs were highly prioritized. Although not a clinical trial, the COS can be used in daily practice for the measurement-based care of patients with PD.

Our study had several limitations. Panel recruitment was insufficient since no Western medical doctors (WMDs) were included (Supplementary Table S4). Since Korea has a parallel medical system in which conventional WMDs and KMDs coexist independently [29], structural conflicts inevitably arise among medical professionals. Conflicts surrounding medical procedures and diagnostic examinations have frequently arisen because WMDs and KMDs must share a fixed national health insurance reimbursement [30]. Recently, conflicts over the reimbursement of HMs have increased, and WMDs have opposed the use and reimbursement of HMs in primary care. Thus, WMDs have negative biases and conflicts of interest regarding herbal treatment. Additionally, WMDs have less understanding of KM because little KM education is provided in WM schools. Therefore, we composed an expert panel of only KMDs specializing in gynecology. As the COS-STAD recommends involving patients in the development process, a semistructured interview with patients is recommended to refine the COS in subsequent studies.

## 5. Conclusions

COS-PD-KM is a core outcome set for evaluating the safety and effectiveness of HM treatment for patients with PD in primary clinics. This COS on PD is the first developed for primary KM care settings and is expected to standardize outcome selection and reporting, aiding in an efficient evaluation of the therapeutic effects of HM for PD. This COS will be used in the HM pilot project, with continuous discussions to increase the degree of completeness and reliability. Further studies are needed that include more relevant stakeholder groups, such as patient representatives and gynecological experts.

## Figures and Tables

**Table 1:** Results of extracted outcomes and effect modifier items of primary dysmenorrhea (PD) in Phase 1.

List of Outcomes and Effect Modifiers	Step 1: Literature Review (10 SRs)	Step 2: Further Reviews on Related Literature	Step 3: PMG Discussions (Outcomes Discussed in Phase 2)
**Outcomes**			
Aspartate transaminase	○	○	○
Alanine transaminase	○	○	○
Blood urea nitrogen	○	○	○
Creatinine	○	○	○
Erythrocyte sedimentation rate	○	○	○
C-reactive protein	○	○	○
Adverse events	○		○
Visual analog scale for pain	○	○	○
Numeric rating scale for pain	○	○	○
Pain duration for one menstrual cycle	○		○
Consumption of painkiller for one menstrual cycle	○		○
Multidimensional Verbal Rating Scale		○	○
Verbal Multidimensional Scoring System		○	○
COX Menstrual Symptom Scale	○		○
McGill Pain Questionnaire	○	○	○
Brief Pain Questionnaire	○		Not specific to PD and similar to NRS
Depression as an associated symptom	○		Discussed that it is more suitable as effect modifier
Scale for quality of life (QoL) (12-Item Short Form Survey or 36-Item Short Form Survey)	○		Difficult to assess in KM primary clinic
Scale for QoL (EuroQol-5 Dimensions-5 Level)	○		○
Satisfaction with treatment	○		○
**Effect modifiers**			
Age		○	○
Onset of primary dysmenorrhea		○	○
Longer and heavier menstrual flow		○	○
Regular/irregular menstrual cycle		○	○
Consumption of drug interaction oral contraceptive pill (within 3 months)		○	○
Use of intrauterine devices		○	○
Use of other drugs		○	○
Age of menarche (early menarche)		○	○
Obesity (including body mass index)		○	○
Shortage of exercise/activities		○	○
Obstetric history (parity/abortion)		○	○
Family history		○	○
Smoking history		○	○
Diet (repeated intake of caffeine, dairy products, omega-3, etc.)		○	○
Psychological factors (anxiety, depression, stress)		○	○

○: The outcome or effect modifer was included.

**Table 2:** Results of the modified Delphi consensus exercise with primary clinicians.

Category	Question	Mean	Median	Degree of Consensus	Degree of Convergence
**Outcomes**	NRS for pain	8.27	9.00	0.89	0.50
	Pain duration	6.73	7.00	0.50	1.75
	Consumption of painkiller	7.09	7.00	0.64	1.25
	Satisfaction of treatment	7.73	8.00	0.88	0.50
	Quality of life (EQ-5D-5 L and EQ-VAS)	5.36	6.00	0.58	1.25
**Effect modifiers** about menstrual/related gynecological history	Age; Longer and heavier menstrual flow; Regular/irregular menstrual cycle; Consumption of drug interaction oral contraceptive pill (within 3 months); Use of intrauterine devices; Use of other drugs; Obesity (including body mass index); Obstetric history (parity/abortion); Family history; Psychological factor (anxiety, depression, stress); Menstrual blood clots	7.27	8.00	0.75	1.00

The CVR, critical value of ≥0.636—suitable for 11 panelists—was judged to suggest consensus among the panelists. A degree of consensus of ≥0.75 and a degree of convergence of ≤0.5 were judged to indicate that agreement among panel experts was achieved. EQ-5D-5 L, EuroQol-5 Dimensions-5; EQ-VAS, EuroQol visual analog scale; NRS, numeric rating scale.

**Table 3:** Results of the final modified Delphi consensus exercise with experts.

Category/Question	Mean	Median	Degree of Consensus	Degree of Convergence
**Outcomes**				
NRS for pain	4	4	1	0
Pain duration for one menstrual cycle	3.25	4	1	0
Consumption of pain killers for one menstrual cycle	3.5	4	0.75	0.5
**Effect modifiers**				
Age	3.2	4	0.75	0.5
Longer and heavier menstrual flow	3.25	4	0.75	0.5
Regular/irregular menstrual cycle	4	4	1	0
Consumption of OCPs (within 3 months)	2.6	3	0	1.5
Use of IUD	3.6	4	0.75	0.5
Use of other drugs	2.4	3	0.33	1
Obesity (including BMI)	2	2	0	1
Obstetric history (parity/abortion)	2.2	4	0.75	0.5
Family history	2.2	3	0.33	1
Psychological factor (NRS of stress intensity)	3.6	4	0.75	0.5
Menstrual blood clots	2.6	3	0	1.5

A degree of consensus of ≥0.75 and a degree of convergence of ≤0.5 were judged to suggest agreement among panel experts. BMI, body mass index; IUD, intrauterine devices; NRS, numeric rating scale; OCP, drug-interacting oral contraceptive pill.

## Supplementary Materials

The following supporting information can be downloaded at https://www.mdpi.com/article/10.3390/ijerph192215321/s1, Table S1. Herbal medicine list covered by national health insurance in South Korea; Table S2. Summary of literature search; Table S3. Characteristics of the original systematic review and meta-analysis on primary dysmenorrhea [10,11,17,31,32,33,34,35,36,37]; Table S4. The reason why we did not include Western medical doctors in the process of developing COS; Figure S1. Flow chart of the selection process.
