# Supplementary material for: Development of the Korean Medicine Core Outcome Set for Primary Dysmenorrhea (COS-PD-KM) for Herbal Medicine Treatment of Primary Dysmenorrhea in Primary Clinics
Source: Int J Environ Res Public Health. 2022 Nov 19;19(22):15321. doi: 10.3390/ijerph192215321 (PMC9690837; doi:10.3390/ijerph192215321)

Table S1. Herbal medicine list covered by national health insurance in South Korea (Korean medicine doctors usually prescribe two packs [120 ml/pack] daily for 10 days to patients with primary dysmenorrhea)

No.	Herbal medicine	Composition (Dose g/pack) *
1	Gamichengsim-tang	Puerariae Radix 7.5 g, Coicis Semen 7.5 g , Dioscoreae Rhizoma 7.5 g, Nelumbinis Semen 7.5 g, Raphani Semen 5.63 g, Phyllostachyos Caulis in Taeniam 5.63 g, Liriopis seu Ophiopogonis Tuber 3.75 g, Platycodonis Radix 3.75 g, Polygalae Radix 3.75 g, Acori Graminei Rhizoma 3.75 g, Zizyphi Semen 3.75 g, Scutellariae Radix 3.75 g, Ligustici Tenuissimi Rhizoma et Radix 3.75 g, Cimicifugae Rhizoma 3.75 g, Angelicae Dahuricae Radix 3.75 g, Longan Arillus 3.75 g
2	Galgeunhaegi-tang_Sasang	Puerariae Radix 11.25 g, Cimicifugae Rhizoma 7.5 g, Scutellariae Radix g, Armeniacae Semen 5.63 g, Zizyphi Semen 3.75 g, Platycodonis Radix 3.75 g, Rhei Radix et Rhizoma 3.75 g, Angelicae Dahuricae Radix 3.75 g, Atractylodis Rhizoma 2.25 g
3	geopung-tang	Zingiberis Rhizoma Recens 11.25 g, Cinnamomi Ramulus, Pinelliae Tuber 7.5 g, Paeoniae Radix 3.75 g, Atractylodis Rhizoma Alba 3.75 g, Citri Unshius Pericarpium 3.75 g, Glycyrrhizae Radix et Rhizoma 3.75 g, Aurantii Fructus Immaturus 3.75 g, Citri Unshius Pericarpium Immaturus 3.75 g, Linderae Radix 3.75 g, Arisaematis Rhizoma 3.75 g, Angelicae Gigantis Radix 3.75 g, Cnidii Rhizoma 3.75 g
4	Gwakhyangjunggi-san_Sasang	Zingiberis Rhizoma Recens 11.25 g, Cinnamomi Ramulus, Pinelliae Tuber 7.5 g, Paeoniae Radix 3.75 g, Atractylodis Rhizoma Alba 3.75 g, Citri Unshius Pericarpium 3.75 g, Glycyrrhizae Radix et Rhizoma 3.75 g, Aurantii Fructus Immaturus 3.75 g, Citri Unshius Pericarpium Immaturus 3.75 g, Linderae Radix 3.75 g, Arisaematis Rhizoma 3.75 g, Angelicae Gigantis Radix 3.75 g, Cnidii Rhizoma 3.75 g
5	Gunggwichongsoyijung-tang	Ginseng Radix 11.25 g, Atractylodis Rhizoma Alba 7.5 g, Zingiberis Rhizoma 7.5 g, Cinnamomi Cortex 7.5 g, Aconiti Lateralis Radix Preparata 7.5 g, Paeoniae Radix 3.75 g, Citri Unshius Pericarpium 3.75 g, Glycyrrhizae Radix et Rhizoma 3.75 g, Cnidii Rhizoma 3.75 g, Angelicae Gigantis Radix 3.75 g, Allii Fistulosi Bulbus 3.75 g, Perillae Folium 3.75 g
6	Doksampalmul-tang	Ginseng Radix 37.5 g, Angelicae Gigantis Radix 5.63 g, Cnidii Rhizoma 3.75 g, Glycyrrhizae Radix et Rhizoma 3.75 g, Astragali Radix 3.75 g, Atractylodis Rhizoma Alba 3.75 g, Paeoniae Radix 3.75 g, Citri Unshius Pericarpium 3.75 g, Zizyphi Fructus 2 g, Zingiberis Rhizoma Recens 1.5 g
7	Mahwangjeongcheon-tang	Ginkgonis Semen 11.25 g, Ephedrae Herba 11.25 g, Armeniacae Semen 5.63 g, Farfarae Flos 3.75 g, Scutellariae Radix 3.75 g, Platycodonis Radix 3.75 g, Liriopis seu Ophiopogonis Tuber 3.75 g, Raphani Semen 3.75 g, Mori Radicis Cortex 3.75 g
8	Moktongdaean-tang	Akebiae Caulis 18.75 g, Rehmanniae Radix Recens 18.75 g, Poria Sclerotium 7.5 g, Alismatis Rhizoma 3.75 g, Plantaginis Semen 3.75 g, Coptidis Rhizoma 3.75 g, Osterici seu Notopterygii Radix et Rhizoma 3.75 g, Schizonepetae Spica 3.75 g, Saposhnikoviae Radix 3.75 g
9	Bojungikgi-tang_Sasang	Ginseng Radix, Astragali Radix 11.25 g, Atractylodis Rhizoma Alba 3.75 g, Angelicae Gigantis Radix 3.75 g, Citri Unshius Pericarpium 3.75 g, Glycyrrhizae Radix et Rhizoma 3.75 g, Perillae Folium 1.88 g, Agastachis Herba 1.88 g, Zingiberis Rhizoma Recens 2.5 g, Zizyphi Fructus 3 g
10	Bopyewon-tang	Liriopis seu Ophiopogonis Tuber 11.25 g, Platycodonis Radix 7.5 g, Schisandrae Fructus 3.75 g, Dioscoreae Rhizoma 3.75 g, Coicis Semen, Raphani Semen 3.75 g
11	Seungyangikgibuja-tang	Ginseng Radix 7.5 g, Cinnamomi Ramulus, Paeoniae Radix, Astragali Radix 7.5 g, Cynanchi Wilfordii Radix 3.75 g, Cinnamomi Cortex 3.75 g, Glycyrrhizae Radix et Rhizoma 3.75 g, Aconiti Lateralis Radix Preparata 3.75 g, Zingiberis Rhizoma Recens 1.5 g, Zizyphi Fructus 2 g
12	Sipyimigwanjung-tang	Cynanchi Wilfordii Radix 3.75 g, Polygoni Multiflori Radix 3.75 g, Zingiberis Rhizoma 3.75 g, Citri Unshius Pericarpium 3.75 g, Citri Unshius Pericarpium Immaturus 3.75 g, Cyperi Rhizoma, Alpiniae Oxyphyllae Fructus 3.75 g, Magnoliae Cortex 3.75 g, Ponciri Fructus Immaturus 3.75 g, Aucklandiae Radix 3.75 g, Arecae Pericarpium 3.75 g, Zingiberis Rhizoma Recens 1.5 g, Zizyphi Fructus 2 g
13	Yangkyuksanwha-tang	Rehmanniae Radix Recens 7.5 g, Lonicerae Folium et Caulis 7.5 g, Forsythiae Fructus 7.5 g, Gardeniae Fructus 3.75 g, Menthae Herba 3.75 g, Anemarrhenae Rhizoma 3.75 g, Gypsum Fibrosum 3.75 g, Saposhnikoviae Radix 3.75 g, Schizonepetae Spica 3.75 g
14	Yeoldahanso-tang	Puerariae Radix 15 g, Scutellariae Radix 7.5 g, Ligustici Tenuissimi Rhizoma et Radix 7.5 g, Raphani Semen 3.75 g, Platycodonis Radix 3.75 g, Cimicifugae Rhizoma 3.75 g, Angelicae Dahuricae Radix 3.75 g
15	Indongdeungjikolpi-tang	Lonicerae Folium et Caulis 15 g, Corni Fructus, Lycii Radicis Cortex 7.5 g, Coptidis Rhizoma, Phellodendri Cortex 3.75 g, Scrophulariae Radix 3.75 g, Sophorae Radix 3.75 g, Rehmanniae Radix Recens 3.75 g, Anemarrhenae Rhizoma 3.75 g, Gardeniae Fructus 3.75 g, Lycii Fructus 3.75 g, Rubi Fructus 3.75 g, Schizonepetae Spica 3.75 g, Saposhnikoviae Radix 3.75 g, Lonicerae Flos 3.75 g
16	Jeoreongchajeonja-tang	Poria Sclerotium 7.5 g, Alismatis Rhizoma 7.5 g, Polyporus, Plantaginis Semen 5.63 g, Osterici seu Notopterygii Radix et Rhizoma 3.75 g, Schizonepetae Spica 3.75 g, Saposhnikoviae Radix 3.75 g, Gypsum Fibrosum 3.75 g, Araliae Continentalis Radix 3.75 g, Anemarrhenae Rhizoma 3.75 g
17	Jowisengchungtang	Coicis Semen 11.25 g, Castaneae Semen 11.25 g, Raphani Semen 5.63 g, Schisandrae Fructus 3.75 g, Acori Graminei Rhizoma 3.75 g, Zizyphi Semen 3.75 g, Polygalae Radix 3.75 g, Longan Arillus 3.75 g, Platycodonis Radix 3.75 g, Ephedrae Herba 3.75 g, Liriopis seu Ophiopogonis Tuber 3.75 g, Asparagi Tuber 3.75 g
18	Cheongsimyeonja-tang	Nelumbinis Semen 7.5 g, Dioscoreae Rhizoma 7.5 g, Asparagi Tuber 3.75 g, Liriopis seu Ophiopogonis Tuber 3.75 g, Polygalae Radix 3.75 g, Acori Graminei Rhizoma 3.75 g, Zizyphi Semen 3.75 g, Longan Arillus 3.75 g, Thujae Semen 3.75 g, Scutellariae Radix 3.75 g, Raphani Semen 3.75 g, Chrysanthemi Indici Flos 1.41 g
19	cheongsimyeolda-tang	Nelumbinis Semen 7.5 g, Dioscoreae Rhizoma, Puerariae Radix 7.5 g, Raphani Semen 3.75 g, Asparagi Tuber 3.75 g, Liriopis seu Ophiopogonis Tuber 3.75 g, Polygalae Radix 3.75 g, Acori Graminei Rhizoma 3.75 g, Zizyphi Semen 3.75 g, Longan Arillus 3.75 g, Thujae Semen 3.75 g, Scutellariae Radix 3.75 g, Cimicifugae Rhizoma 3.75 g, Ligustici Tenuissimi Rhizoma et Radix 3.75 g, Angelicae Dahuricae Radix 3.75 g, Platycodonis Radix 3.75 g, Chrysanthemi Indici Flos 1.88 g

**20**	Palmulgunja-tang	Ginseng Radix 3.75 g, Astragali Radix 3.75 g, Atractylodis Rhizoma Alba 3.75 g, Angelicae Gigantis Radix 3.75 g, Cnidii Rhizoma 3.75 g, Paeoniae Radix 3.75 g, Citri Unshius Pericarpium 3.75 g, Glycyrrhizae Radix et Rhizoma 3.75 g, Zingiberis Rhizoma Recens 2.5 g, Zizyphi Fructus 3 g
**21**	Handayeolso-tang	Coicis Semen, Castaneae Semen 11.25 g, Raphani Semen 7.5 g, Liriopis seu Ophiopogonis Tuber 3.75 g, Platycodonis Radix 3.75 g, Scutellariae Radix 3.75 g, Armeniacae Semen 3.75 g, Ephedrae Herba 3.75 g
**22**	Hyangsayangwi-tang	Ginseng Radix 3.75 g, Atractylodis Rhizoma Alba 3.75 g, Paeoniae Radix 3.75 g, Pinelliae Tuber 3.75 g, Cyperi Rhizoma 3.75 g, Citri Unshius Pericarpium 3.75 g, Zingiberis Rhizoma 3.75 g, Crataegi Fructus 3.75 g, Amomi Fructus 3.75 g, Amomi Fructus Rotundus 3.75 g, Glycyrrhizae Radix et Rhizoma 3.75 g
**23**	Hyungbangdojuck-san	Rehmanniae Radix Recens 11.25 g, Akebiae Caulis 7.5 g, Scrophulariae Radix 5.63 g, Trichosanthis Semen 5.63 g, Peucedani Radix 3.75 g, Osterici seu Notopterygii Radix et Rhizoma 3.75 g, Araliae Continentalis Radix 3.75 g, Schizonepetae Spica 3.75 g, Saposhnikoviae Radix 3.75 g
**24**	Hyungbangsabaek-san	Rehmanniae Radix Recens 11.25 g, Poria Sclerotium, Alismatis Rhizoma 7.5 g, Gypsum Fibrosum 3.75 g, Anemarrhenae Rhizoma 3.75 g, Osterici seu Notopterygii Radix et Rhizoma 3.75 g, Araliae Continentalis Radix 3.75 g, Schizonepetae Spica 3.75 g, Saposhnikoviae Radix 3.75 g
**25**	Hyeungbangjihwang-tang	Rehmanniae Radix Preparata 11.25 g, Corni Fructus, Poria Sclerotium 7.5 g, Alismatis Rhizoma 7.5 g, Plantaginis Semen 3.75 g, Osterici seu Notopterygii Radix et Rhizoma 3.75 g, Araliae Continentalis Radix 3.75 g, Schizonepetae Spica 3.75 g, Saposhnikoviae Radix 3.75 g, Moutan Radicis Cortex 3.75 g
**26**	Hyeongbangpaedoksan_Sasang	Osterici seu Notopterygii Radix et Rhizoma 3.75 g, Araliae Continentalis Radix 3.75 g, Bupleuri Radix 3.75 g, Peucedani Radix 3.75 g, Schizonepetae Spica 3.75 g, Saposhnikoviae Radix 3.75 g, Poria Sclerotium 3.75 g, Rehmanniae Radix Recens 3.75 g, Lycii Radicis Cortex 3.75 g, Plantaginis Semen 3.75 g
**27**	Hwalsuckgosam-tang	Alismatis Rhizoma 7.5 g, Poria Sclerotium 7.5 g, Talcum, Sophorae Radix 7.5 g, Coptidis Rhizoma 3.75 g, Phellodendri Cortex 3.75 g, Osterici seu Notopterygii Radix et Rhizoma 3.75 g, Araliae Continentalis Radix 3.75 g, Schizonepetae Spica 3.75 g, Saposhnikoviae Radix 3.75 g
**28**	hwanglyeoncheongjang-tang	Rehmanniae Radix Recens 15 g, Akebiae Caulis 7.5 g, Alismatis Rhizoma 7.5 g, Poria Sclerotium 7.5 g, Polyporus 3.75 g, Plantaginis Semen 3.75 g, Coptidis Rhizoma 3.75 g, Osterici seu Notopterygii Radix et Rhizoma 3.75 g, Saposhnikoviae Radix 3.75 g
**29**	Gamisoyo-san_Pattern Identification	Moutan Radicis Cortex 5.63 g, Atractylodis Rhizoma Alba 5.63 g, Paeoniae Radix 3.75 g, Persicae Semen 3.75 g, Fritillariae Thunbergii Bulbus 3.75 g, Gardeniae Fructus 3 g, Scutellariae Radix 3 g, Platycodonis Radix 2.63 g, Citri Unshius Pericarpium Immaturus 1.88 g, Glycyrrhizae Radix et Rhizoma 1.13 g
**30**	Galgeun-tang_Pattern Identification	Puerariae Radix 7.5 g, Cimicifugae Rhizoma 3.75 g, Gentianae Macrophyllae Radix 3.75 g, Schizonepetae Spica 3.75 g, Paeoniae Radix 3.75 g, Perillae Folium 3 g, Angelicae Dahuricae Radix 3 g, Glycyrrhizae Radix et Rhizoma 1.88 g, Zingiberis Rhizoma Recens 1.5 g
**31**	Guibi-tang	Angelicae Gigantis Radix 3.75 g, Longan Arillus 3.75 g, Zizyphi Semen 3.75 g, Polygalae Radix 3.75 g, Ginseng Radix 3.75 g, Astragali Radix 3.75 g, Atractylodis Rhizoma Alba 3.75 g, Poria Sclertum Cum Pini Radix 3.75 g, Aucklandiae Radix 1.88 g, Glycyrrhizae Radix et Rhizoma 1.13 g, Zingiberis Rhizoma Recens 2.5 g, Zizyphi Fructus 2 g
**32**	Daeseunggi-tang	Rhei Radix et Rhizoma 15 g, Magnoliae Cortex 7.5 g, Ponciri Fructus Immaturus 7.5 g, Natrii Sulfas 7.5 g
**33**	Banchong-san	Atractylodis Rhizoma 3.75 g, Glycyrrhizae Radix et Rhizoma 3.75 g, Sparganii Rhizoma 2.82 g, Curcumae Rhizoma 2.82 g, Poria Sclerotium 2.82 g, Citri Unshius Pericarpium Immaturus 2.82 g, Amomi Fructus 1.88 g, Syzygii Flos 1.88 g, Arecae Semen 1.88 g, Corydalis Tuber 1.41 g, Cinnamomi Cortex 1.41 g, Zingiberis Rhizoma 1.41 g, Allii Fistulosi Bulbus 1.63 g
**34**	Banhabakchulchunma-tang	Pinelliae Tuber 5.63 g, Citri Unshius Pericarpium 5.63 g, Hordei Fructus Germinatus 5.63 g, Atractylodis Rhizoma Alba 3.75 g, Massa Medicata Fermentata 3.75 g, Atractylodis Rhizoma 1.88 g, Ginseng Radix 1.88 g, Astragali Radix 1.88 g, Gastrodiae Rhizoma 1.88 g, Poria Sclerotium 1.88 g, Alismatis Rhizoma 1.88 g, Zingiberis Rhizoma 1.13 g, Phellodendri Cortex 0.75 g, Zingiberis Rhizoma Recens 2.5 g
**35**	Banhasasim-tang	Pinelliae Tuber 7.5 g, Scutellariae Radix 5.63 g, Ginseng Radix 5.63 g, Glycyrrhizae Radix et Rhizoma 5.63 g, Zingiberis Rhizoma 3.75 g, Coptidis Rhizoma 1.88 g
**36**	Bangpuangtongseoung-san	Talcum 6.38 g, Glycyrrhizae Radix et Rhizoma 4.5 g, Gypsum Fibrosum 2.63 g, Scutellariae Radix 2.63 g, Platycodonis Radix 2.63 g, Saposhnikoviae Radix 1.69 g, Cnidii Rhizoma 1.69 g, Angelicae Gigantis Radix 1.69 g, Paeoniae Radix 1.69 g, Rhei Radix et Rhizoma 1.69 g, Ephedrae Herba 1.69 g, Menthae Herba 1.69 g, Forsythiae Fructus 1.69 g, Natrii Sulfas 1.69 g, Schizonepetae Spica 1.32 g, Atractylodis Rhizoma Alba 1.32 g, Gardeniae Fructus 1.32 g
**37**	Baekho-tang	Gypsum Fibrosum 18.75 g, Anemarrhenae Rhizoma 7.5 g, Glycyrrhizae Radix et Rhizoma 2.63 g, Oryzae Semen 100 g
**38**	Bulhwangeumjeonggi-san	Atractylodis Rhizoma 7.5 g, Magnoliae Cortex 3.75 g, Citri Unshius Pericarpium 3.75 g, Agastachis Herba 3.75 g, Pinelliae Tuber 3.75 g, Glycyrrhizae Radix et Rhizoma 3.75 g, Zingiberis Rhizoma Recens 1.5 g, Zizyphi Fructus 2 g
**39**	Sayook-tang	Rehmanniae Radix Preparata 15 g, Dioscoreae Rhizoma 7.5 g, Corni Fructus 7.5 g, Poria Sclerotium 5.63 g, Moutan Radicis Cortex 5.63 g, Alismatis Rhizoma 5.63 g, Paeoniae Radix 4.69 g, Cnidii Rhizoma 4.69 g, Angelicae Gigantis Radix 4.69 g
**40**	Sabaek-san	Mori Radicis Cortex 7.5 g, Lycii Radicis Cortex 7.5 g, Anemarrhenae Rhizoma 3.75 g, Fritillariae Thunbergii Bulbus 3.75 g, Liriopis seu Ophiopogonis Tuber 3.75 g, Rehmanniae Radix Recens 3.75 g, Platycodonis Radix 3.75 g, Gardeniae Fructus 3.75 g, Glycyrrhizae Radix et Rhizoma 3.75 g
**41**	Sayeok-san	Bupleuri Radix 5.63 g, Paeoniae Radix 5.63 g, Ponciri Fructus Immaturus 5.63 g, Glycyrrhizae Radix et Rhizoma 5.63 g
**42**	Samryungbeakchul-san	Ginseng Radix 11.25 g, Atractylodis Rhizoma Alba 11.25 g, Poria Sclerotium 11.25 g, Dioscoreae Rhizoma 11.25 g, Glycyrrhizae Radix et Rhizoma 11.25 g, Coicis Semen 5.63 g, Nelumbinis Semen 5.63 g, Platycodonis Radix 5.63 g, Amomi Fructus 5.63 g, Dolichoris Semen 5.63 g
**43**	Sogunjung-tang	Oryzae Gluten 30 g, Paeoniae Radix 11.25 g, Zizyphi Fructus 8, Cinnamomi Ramulus 5.63 g, Zingiberis Rhizoma Recens 2.5 g, Glycyrrhizae Radix et Rhizoma 5.63 g
**44**	Soshiho-tang	Bupleuri Radix 11.25 g, Scutellariae Radix 7.5 g, Ginseng Radix, Pinelliae Tuber 3.75 g, Glycyrrhizae Radix et Rhizoma 1.88 g, Zingiberis Rhizoma Recens 1.5 g, Zizyphi Fructus 2 g
**45**	Sojaganggi-tang	Pinelliae Tuber, Perillae Fructus 3.75 g, Cinnamomi Cortex 3.75 g, Citri Unshius Pericarpium 2.82 g, Angelicae Gigantis Radix 1.88 g, Peucedani Radix 1.88 g, Magnoliae Cortex 1.88 g, Glycyrrhizae Radix et Rhizoma 1.88 g, Perillae Folium 2.44 g, Zingiberis Rhizoma Recens 1.5 g, Zizyphi Fructus 2 g

**46**	Yanggyeok-san	Forsythiae Fructus 7.5 g, Rhei Radix et Rhizoma 3.75 g, Natrii Sulfas 3.75 g, Glycyrrhizae Radix et Rhizoma 3.75 g, Menthae Herba 1.88 g, Scutellariae Radix 1.88 g, Gardeniae Fructus 1.88 g
**47**	Yeogwag-tang	Elsholtziae Herba 7.5 g, Atractylodis Rhizoma 7.5 g, Agastachis Herba, Perillae Folium 5.63 g, Magnoliae Cortex 3.75 g, Citri Unshius Pericarpium 3.75 g, Cyperi Rhizoma 3.75 g, Amomi Fructus 3.75 g, Amomi Tsao-ko Fructus 3.75 g, Crataegi Fructus 3.75 g, Hordei Fructus Germinatus 3.75 g, Dolichoris Semen 3.75 g, Glycyrrhizae Radix et Rhizoma 2.63 g, Pinelliae Tuber 1.88 g, Arecae Pericarpium 1.88 g, Angelicae Dahuricae Radix 1.88 g, Poria Sclerotium 1.88 g, Zingiberis Rhizoma Recens 1.5 g
**48**	Oyaksungi-san	Ephedrae Herba 5.63 g, Citri Unshius Pericarpium, Linderae Radix 5.63 g, Cnidii Rhizoma 3.75 g, Angelicae Dahuricae Radix 3.75 g, Batryticatus Bombyx 3.75 g, Aurantii Fructus Immaturus 3.75 g, Platycodonis Radix 3.75 g, Zingiberis Rhizoma 1.88 g, Glycyrrhizae Radix et Rhizoma 1.13 g, Zingiberis Rhizoma Recens 1.5 g, Zizyphi Fructus 2 g
**49**	Ojeok-san	Atractylodis Rhizoma 7.5 g, Ephedrae Herba 3.75 g, Citri Unshius Pericarpium 3.75 g, Magnoliae Cortex 3 g, Platycodonis Radix 3 g, Aurantii Fructus Immaturus 3 g, Angelicae Gigantis Radix 3 g, Zingiberis Rhizoma 3 g, Paeoniae Radix 3 g, Poria Sclerotium 3 g, Cnidii Rhizoma 2.63 g, Angelicae Dahuricae Radix 2.63 g, Pinelliae Tuber 2.63 g, Cinnamomi Cortex 2.63 g, Glycyrrhizae Radix et Rhizoma 2.25 g, Zingiberis Rhizoma Recens 1.5 g
**50**	Ondam-tang_Pattern Identification	Pinelliae Tuber 7.5 g, Citri Unshius Pericarpium, Poria Sclerotium 7.5 g, Ponciri Fructus Immaturus 7.5 g, Phyllostachyos Caulis in Taeniam 3.75 g, Glycyrrhizae Radix et Rhizoma 1.88 g, Zingiberis Rhizoma Recens 2.5 g, Zizyphi Fructus 2 g
**51**	Wiryeong-tang	Atractylodis Rhizoma 5.63 g, Magnoliae Cortex 5.63 g, Citri Unshius Pericarpium 5.63 g, Polyporus 5.63 g, Alismatis Rhizoma 5.63 g, Atractylodis Rhizoma Alba 5.63 g, Poria Sclerotium, Paeoniae Radix 5.63 g, Cinnamomi Cortex 2.82 g, Glycyrrhizae Radix et Rhizoma 2.82 g, Zingiberis Rhizoma Recens 2.5 g, Zizyphi Fructus 3 g
**52**	YukmihabSaengmaek-san	Rehmanniae Radix Preparata 15 g, Dioscoreae Rhizoma, Corni Fructus Liriopis seu Ophiopogonis Tuber 7.5 g, Poria Sclerotium 5.63 g, Moutan Radicis Cortex 5.63 g, Alismatis Rhizoma 5.63 g, Ginseng Radix 3.75 g, Schisandrae Fructus 3.75 g
**53**	Yukwool-tang	Cyperi Rhizoma 7.5 g, Cnidii Rhizoma 9.38 g, Atractylodis Rhizoma 9.38 g, Citri Unshius Pericarpium 3.75 g, Pinelliae Tuber 3.75 g, Poria Sclerotium 2.63 g, Gardeniae Fructus 2.63 g, Amomi Fructus 1.88 g, Glycyrrhizae Radix et Rhizoma 1.88 g
**54**	Yijung-tang	Ginseng Radix 7.5 g, Atractylodis Rhizoma Alba 7.5 g, Zingiberis Rhizoma 7.5 g, Glycyrrhizae Radix et Rhizoma 3.75 g
**55**	IjinSamul-tang	Pinelliae Tuber 7.5 g, Rehmanniae Radix Preparata 4.69 g, Paeoniae Radix 4.69 g, Cnidii Rhizoma 4.69 g, Angelicae Gigantis Radix 4.69 g, Citri Unshius Pericarpium 3.75 g, Poria Sclerotium 3.75 g, Glycyrrhizae Radix et Rhizoma 1.88 g, Zingiberis Rhizoma Recens 1.5 g
**56**	Insamyangyung-tang	Paeoniae Radix 7.5 g, Angelicae Gigantis Radix 3.75 g, Ginseng Radix 3.75 g, Atractylodis Rhizoma Alba 3.75 g, Astragali Radix 3.75 g, Cinnamomi Cortex 3.75 g, Citri Unshius Pericarpium 3.75 g, Glycyrrhizae Radix et Rhizoma 3.75 g, Rehmanniae Radix Preparata 2.82 g, Schisandrae Fructus 2.82 g, Saposhnikoviae Radix 2.82 g, Polygalae Radix 1.88 g, Zingiberis Rhizoma Recens 1.5 g, Zizyphi Fructus 2 g
**57**	Insamyangwi-tang	Atractylodis Rhizoma 5.63 g, Citri Unshius Pericarpium 4.69 g, Magnoliae Cortex 4.69 g, Pinelliae Tuber 4.69 g, Poria Sclerotium 3.75 g, Agastachis Herba 3.75 g, Ginseng Radix 1.88 g, Amomi Tsao-ko Fructus 1.88 g, Glycyrrhizae Radix et Rhizoma 1.88 g, Zizyphi Fructus 2 g, Zingiberis Rhizoma Recens 1.5 g, Mume Fructus 1 g
**58**	Jaeumganghwa-tang	Paeoniae Radix 4.88 g, Angelicae Gigantis Radix 4.5 g, Rehmanniae Radix Preparata 3.75 g, Liriopis seu Ophiopogonis Tuber 3.75 g, Atractylodis Rhizoma Alba 3.75 g, Rehmanniae Radix Recens 3 g, Citri Unshius Pericarpium 2.63 g, Anemarrhenae Rhizoma 1.88 g, Phellodendri Cortex 1.88 g, Glycyrrhizae Radix et Rhizoma 1.88 g, Zingiberis Rhizoma Recens 1.5 g, Zizyphi Fructus 2 g
**59**	Jeongjeongamiijin-tang	Crataegi Fructus 3.75 g, Cyperi Rhizoma 3.75 g, Pinelliae Tuber 3.75 g, Cnidii Rhizoma 3.75 g, Atractylodis Rhizoma Alba 3.75 g, Atractylodis Rhizoma 3.75 g, Citri Unshius Pericarpium 3.75 g, Poria Sclerotium 3.75 g, Massa Medicata Fermentata 3.75 g, Amomi Fructus 3.75 g, Hordei Fructus Germinatus 3.75 g, Glycyrrhizae Radix et Rhizoma 3.75 g, Zingiberis Rhizoma Recens 2.5 g, Zizyphi Fructus 3 g
**60**	Jukyeopseokgo-tang	Gypsum Fibrosum 15 g, Ginseng Radix 7.5 g, Liriopis seu Ophiopogonis Tuber 5.63 g, Pinelliae Tuber 3.75 g, Glycyrrhizae Radix et Rhizoma 2.63 g
**61**	Jinmu-tang	Poria Sclerotium 11.25 g, Paeoniae Radix 11.25 g, Aconiti Lateralis Radix Preparata 11.25 g, Atractylodis Rhizoma Alba 7.5 g, Zingiberis Rhizoma Recens 2.5 g
**62**	Cheongnijagam-tang	Rehmanniae Radix Preparata 2.63 g, Rehmanniae Radix 2.63 g, Asparagi Tuber 2.63 g, Liriopis seu Ophiopogonis Tuber 2.63 g, Angelicae Gigantis Radix 2.63 g, Paeoniae Radix 2.63 g, Corni Fructus 2.63 g, Dioscoreae Rhizoma 2.63 g, Poria Sclerotium 2.63 g, Atractylodis Rhizoma Alba 2.63 g, Moutan Radicis Cortex 1.88 g, Alismatis Rhizoma 1.88 g, Phellodendri Cortex 1.88 g, Anemarrhenae Rhizoma 1.88 g, Glycyrrhizae Radix et Rhizoma 1.88 g
**63**	Chungseoikgi-tang	Atractylodis Rhizoma 5.63 g, Astragali Radix, Cimicifugae Rhizoma 3.75 g, Ginseng Radix 1.88 g, Atractylodis Rhizoma Alba 1.88 g, Citri Unshius Pericarpium 1.88 g, Massa Medicata Fermentata 1.88 g, Alismatis Rhizoma 1.88 g, Phellodendri Cortex 1.13 g, Angelicae Gigantis Radix 1.13 g, Puerariae Radix 1.13 g, Citri Unshius Pericarpium Immaturus 1.13 g, Liriopis seu Ophiopogonis Tuber 1.13 g, Glycyrrhizae Radix et Rhizoma 1.13 g
**64**	Palmul-tang	Ginseng Radix 4.5 g, Atractylodis Rhizoma Alba 4.5 g, Poria Sclerotium 4.5 g, Glycyrrhizae Radix et Rhizoma 4.5 g, Rehmanniae Radix Preparata 4.5 g, Paeoniae Radix 4.5 g, Cnidii Rhizoma 4.5 g, Angelicae Gigantis Radix 4.5 g
**65**	Paljeong-san	Talcum 3.75 g, Plantaginis Semen 3.75 g , Dianthi Herba 3.75 g , Glycyrrhizae Radix et Rhizoma 3.75 g , Gardeniae Fructus 3.75 g, Junci Medulla 3.75 g, Rhei Radix et Rhizoma 3.75 g, Akebiae Caulis 3.75 g, Polygoni Avicularis Herba 3.75 g
**66**	Hyangsayukgunja-tang	Cyperi Rhizoma 3.75 g, Atractylodis Rhizoma Alba 3.75 g, Poria Sclerotium 3.75 g, Pinelliae Tuber 3.75 g, Citri Unshius Pericarpium 3.75 g, Amomi Fructus Rotundus 3.75 g, Magnoliae Cortex 3.75 g, Amomi Fructus 1.88 g, Ginseng Radix 1.88 g, Aucklandiae Radix 1.88 g, Alpiniae Oxyphyllae Fructus 1.88 g, Glycyrrhizae Radix et Rhizoma 1.88 g, Zingiberis Rhizoma Recens 1.5 g, Zizyphi Fructus 2 g
**67**	Hyangsapyeongwi-san	Atractylodis Rhizoma 7.5 g, Citri Unshius Pericarpium 3.75 g, Cyperi Rhizoma 3.75 g, Ponciri Fructus Immaturus 3 g, Agastachis Herba 3 g, Magnoliae Cortex, Amomi Fructus 2.63 g, Aucklandiae Radix 1.88 g, Glycyrrhizae Radix et Rhizoma 1.88 g, Zingiberis Rhizoma Recens 1.5 g
**68**	Hwanggigyejiomul-tang	Zingiberis Rhizoma Recens 11.25 g, Zizyphi Fructus 8 g, Astragali Radix 5.63 g, Paeoniae Radix 5.63 g, Cinnamomi Ramulus 5.63 g

**69**	hwangryeon-tang	Pinelliae Tuber 11.25 g, Zizyphi Fructus 8 g, Coptidis Rhizoma 5.63 g, Zingiberis Rhizoma 5.63 g, Cinnamomi Ramulus 5.63 g, Glycyrrhizae Radix et Rhizoma 5.63 g, Ginseng Radix 3.75 g
**70**	Hwangryunhaedok-tang	Coptidis Rhizoma 4.69 g, Scutellariae Radix 4.69 g, Phellodendri Cortex 4.69 g, Gardeniae Fructus 4.69 g
**71**	Kagamojeok-san	Cyperi Rhizoma 7.5 g, Atractylodis Rhizoma 7.5 g, Citri Unshius Pericarpium 3.75 g, Patriniae Radix 3.75 g, Magnoliae Cortex 3 g, Platycodonis Radix 3 g, Aurantii Fructus Immaturus 3 g, Poria Sclerotium 3 g, Angelicae Gigantis Radix 3 g, Paeoniae Radix 3 g, Sparganii Rhizoma 3 g, Curcumae Rhizoma 3 g, Persicae Semen 3 g, Carthami Flos 3 g, Sappan Lignum 3 g, Corydalis Tuber 3 g, Zingiberis Rhizoma 3 g, Cassiae Cortex Interior 2.25 g, Pinelliae Tuber 2.25 g, Cnidii Rhizoma 2.25 g, Angelicae Dahuricae Radix 2.25 g, Glycyrrhizae Radix et Rhizoma 2.25 g, Zingiberis Rhizoma Recens 1.5 g
**72**	Kagamjogyeong-tang	Cyperi Rhizoma 7.5 g, Angelicae Gigantis Radix 7.5 g, Longan Arillus 7.5 g, Atractylodis Rhizoma Alba 5.63 g, Rehmanniae Radix Preparata 5.63 g, Linderae Radix 5.63 g, Citri Unshius Pericarpium 5.63 g, Cnidii Rhizoma 3.75 g, Paeoniae Radix 3.75 g, Moutan Radicis Cortex 3.75 g, Aucklandiae Radix 3.75 g, Zingiberis Rhizoma 3.75 g, Foeniculi Fructus 3.75 g, Crataegi Fructus 3.75 g, Corydalis Tuber 1.88 g, Carthami Flos 1.88 g, Glycyrrhizae Radix et Rhizoma 1.88 g, Cinnamomi Cortex 1.88 g
**73**	GamidanggwiJakyak-tang	Paeoniae Radix 11.25 g, Poria Sclerotium 7.5 g, Atractylodis Rhizoma Alba 7.5 g, Alismatis Rhizoma 7.5 g, Angelicae Gigantis Radix 7.5 g, Cnidii Rhizoma 2.82 g, Agastachis Herba 2.82 g, Ponciri Fructus Immaturus 2.82 g, Platycodonis Radix 2.82 g, Aucklandiae Radix 2.82 g, Amomi Fructus 2.82 g, Glycyrrhizae Radix et Rhizoma 2.82 g
**74**	Gamisajin-tang	Persicae Semen 11.25 g, Angelicae Gigantis Radix 7.5 g, Paeoniae Radix, Cnidii Rhizoma 7.5 g, Foeniculi Fructus 3.75 g, Cinnamomi Cortex 3.75 g, Aurantii Fructus Immaturus 3.75 g, Atractylodis Rhizoma 3.75 g, Salviae Miltiorrhizae Radix 3.75 g, Rehmanniae Radix Recens 3.75 g, Carthami Flos 3.75 g, Glycyrrhizae Radix et Rhizoma 3.75 g, Corydalis Tuber 3.75 g, Rhei Radix et Rhizoma 3.75 g, Linderae Radix 3.75 g
**75**	Gamisachil-tang	Pinelliae Tuber 7.5 g, Poria Sclerotium 4.5 g, Magnoliae Cortex 4.5 g, Poria Sclertum Cum Pini Radix 3 g, Perillae Folium 3 g, Polygalae Radix 1.88 g, Glycyrrhizae Radix et Rhizoma 1.88 g, Acori Graminei Rhizoma 1.88 g, Zingiberis Rhizoma Recens 2.5 g, Zizyphi Fructus 2 g
**76**	Keukhachukeo-tang	Paeoniae Radix 15 g, Angelicae Gigantis Radix 10 g, Cnidii Rhizoma 10 g, Persicae Semen 10 g, Aurantii Fructus Immaturus 10 g, Corydalis Tuber 10 g, Linderae Radix 10 g, Trogopterorum Faeces 10 g, Glycyrrhizae Radix et Rhizoma 10 g, Moutan Radicis Cortex 10 g, Carthami Flos, Eucommiae Cortex 10 g, Cyperi Rhizoma 10 g, Paeoniae Radix 10 g
**77**	Gyejibongnyeong-hwankagam	Cinnamomi Ramulus 5.63 g, Poria Sclerotium 5.63 g, Moutan Radicis Cortex 5.63 g, Persicae Semen 5.63 g, Paeoniae Radix 5.63 g, Coicis Semen 5.63 g, Lonicerae Flos 3.75 g, Smilacis Rhizoma 2.82 g, Leonuri Herba 2.35 g, Corydalis Tuber 2.35 g
**78**	Gyoaesamul-tang	Rehmanniae Radix Preparata 3.75 g, Angelicae Gigantis Radix 3.75 g, Cnidii Rhizoma 3.75 g, Paeoniae Radix 3.75 g, Atractylodis Rhizoma Alba 3.75 g, Scutellariae Radix 3.75 g, Artemisiae Argyi Folium 3.75 g, Amomi Fructus 3.75 g, Cyperi Rhizoma 3.75 g, Asini Corii Colla 3.75 g, Oryzae Semen 9.38 g
**79**	Gunggi-tangkagam	Angelicae Gigantis Radix 18.75 g, Cnidii Rhizoma 18.75 g, Ginseng Radix 3.75 g, Astragali Radix 3.75 g, Paeoniae Radix 3.75 g, Cyperi Rhizoma 3.75 g
**80**	Guichulpajing-tang	Cyperi Rhizoma 5.63 g, Sparganii Rhizoma 3.75 g, Curcumae Rhizoma 3.75 g, Paeoniae Radix 3.75 g, Paeoniae Radix 3.75 g, Angelicae Gigantis Radix 3.75 g, Citri Unshius Pericarpium Immaturus 3.75 g, Linderae Radix 2.63 g, Carthami Flos, Sappan Lignum, Cinnamomi Cortex 1.88 g
**81**	Dangguijakyak-san	Cnidii Rhizoma, Paeoniae Radix 15 g, Angelicae Gigantis Radix 10 g, Paeoniae Radix 10 g, Poria Sclerotium 10 g, Alismatis Rhizoma 10 g, Atractylodis Rhizoma Alba 10 g, Linderae Radix 10 g, Cyperi Rhizoma 10 g, Corydalis Tuber 10 g, Glycyrrhizae Radix et Rhizoma 10 g
**82**	Daeyeongondam-tang	Rehmanniae Radix Preparata 11.25 g, Cyperi Rhizoma 8.44 g, Angelicae Gigantis Radix 7.5 g, Lycii Fructus 7.5 g, Eucommiae Cortex 7.5 g, Achyranthis Radix 5.63 g, Citri Unshius Pericarpium 4.22 g, Cinnamomi Cortex 3.75 g, Glycyrrhizae Radix et Rhizoma 3.75 g, Pinelliae Tuber 2.82 g, Ponciri Fructus Immaturus 2.82 g, Phyllostachyos Caulis in Taeniam 2.82 g, Ginseng Radix 2.25 g, Platycodonis Radix 2.25 g, Poria Sclerotium 2.25 g, Bupleuri Radix 2.11 g, Liriopis seu Ophiopogonis Tuber 2.11 g, Zingiberis Rhizoma Recens 1.5 g, Zizyphi Fructus 2 g
**83**	Daeyeongjeon	Rehmanniae Radix Preparata 11.25 g, Angelicae Gigantis Radix 7.5 g, Lycii Fructus 7.5 g, Eucommiae Cortex 7.5 g, Achyranthis Radix 5.63 g, Cinnamomi Cortex 3.75 g, Glycyrrhizae Radix et Rhizoma 3.75 g
**84**	Doin-tang	Persicae Semen 18.75 g, Sappan Lignum 18.75 g, Rehmanniae Radix Recens 18.75 g, Tabanus 3 g, Hirudo 50 g
**85**	hongsamul-tang	Persicae Semen 15 g, Angelicae Gigantis Radix 15 g, Cnidii Rhizoma 15 g, Rehmanniae Radix Preparata 15 g, Carthami Flos 15 g, Paeoniae Radix 15 g, Curcumae Radix 15 g, Bupleuri Radix 15 g
**86**	Boyanghwano-tang	Astragali Radix 56.25 g, Angelicae Gigantis Radix 7.5 g, Paeoniae Radix 5.63 g, Lumbricus 3.75 g, Cnidii Rhizoma 3.75 g, Persicae Semen 3.75 g, Carthami Flos 3.75 g
**87**	Bojungikgi-tang	Astragali Radix 5.63 g, Ginseng Radix 3.75 g, Atractylodis Rhizoma Alba 3.75 g, Glycyrrhizae Radix et Rhizoma 3.75 g, Angelicae Gigantis Radix 1.88 g, Citri Unshius Pericarpium 1.88 g, Cimicifugae Rhizoma 1.13 g, Bupleuri Radix 1.13 g
**88**	Samuljogyeong-tang	Cyperi Rhizoma 3.75 g, Aurantii Fructus Immaturus 2.82 g, Bupleuri Radix 2.82 g, Cnidii Rhizoma 2.82 g, Angelicae Gigantis Radix 2.82 g, Scutellariae Radix 2.82 g, Paeoniae Radix 2.82 g, Carthami Flos 1.88 g, Corydalis Tuber 1.88 g, Curcumae Rhizoma 1.88 g, Rehmanniae Radix Preparata 1.88 g, Foeniculi Fructus 1.88 g, Glycyrrhizae Radix et Rhizoma 1.88 g, Sparganii Rhizoma 1.88 g, Amomi Fructus 1.88 g, Angelicae Dahuricae Radix 1.88 g, Citri Unshius Pericarpium 1.88 g, Citri Unshius Pericarpium Immaturus 1.88 g, Atractylodis Rhizoma Alba 1.88 g
**89**	Seongyu-tang	Rehmanniae Radix Preparata 7.5 g , Rehmanniae Radix Recens 7.5 g , Cnidii Rhizoma 7.5 g , Ginseng Radix 7.5 g, Angelicae Gigantis Radix 3.75 g, Astragali Radix 3.75 g
**90**	Seganmeongmok-tang	Angelicae Gigantis Radix 1.88 g, Cnidii Rhizoma 1.88 g, Paeoniae Radix 1.88 g, Rehmanniae Radix Recens 1.88 g, Coptidis Rhizoma 1.88 g, Scutellariae Radix 1.88 g, Gardeniae Fructus 1.88 g, Gypsum Fibrosum 1.88 g, Forsythiae Fructus 1.88 g, Saposhnikoviae Radix 1.88 g, Schizonepetae Spica 1.88 g, Menthae Herba 1.88 g, Osterici seu Notopterygii Radix et Rhizoma 1.88 g, Viticis Fructus 1.88 g, Chrysanthemi Indici Flos 1.88 g, Tribuli Fructus 1.88 g, Cassiae Semen 1.88 g, Platycodonis Radix 1.88 g, Glycyrrhizae Radix et Rhizoma 1.88 g
**91**	Sobokchuko-tang	Angelicae Gigantis Radix 20 g, Cnidii Rhizoma 20 g, Foeniculi Fructus 15 g, Polygalae Radix 15 g, Cinnamomi Ramulus 15 g, Paeoniae Radix 15 g, Cyperi Rhizoma 15 g, Loranthi Ramulus Et Folium 15 g, Leonuri Herba 15 g, Glycyrrhizae Radix et Rhizoma 10 g, Achyranthis Radix 10 g, Zingiberis Rhizoma 5 g

**92**	Soyo-san	Angelicae Gigantis Radix 15 g, Bupleuri Radix 15 g, Achyranthis Radix 15 g, Corydalis Tuber 15 g, Rehmanniae Radix Recens 10 g, Persicae Semen 10 g, Carthami Flos 10 g, Aurantii Fructus Immaturus 10 g, Paeoniae Radix 10 g, Glycyrrhizae Radix et Rhizoma 10 g, Cnidii Rhizoma 10 g, Platycodonis Radix 10 g
**93**	Sopunghwalhyeol-tang	Angelicae Gigantis Radix 3.75 g, Cnidii Rhizoma, Clematidis Radix 3.75 g, Angelicae Dahuricae Radix 3.75 g, Sinomeni Caulis et Rhizoma 3.75 g, Phellodendri Cortex 3.75 g, Arisaematis Rhizoma 3.75 g, Atractylodis Rhizoma 3.75 g, Osterici seu Notopterygii Radix et Rhizoma 3.75 g, Cinnamomi Ramulus 3.75 g, Carthami Flos 1.13 g, Zingiberis Rhizoma Recens 2.5 g
**94**	Sintongchukea-tang	Persicae Semen 8.44 g, Carthami Flos 8.44 g, Angelicae Gigantis Radix 8.44 g, Achyranthis Radix 8.44 g, Gentianae Macrophyllae Radix 8.44 g, Osterici seu Notopterygii Radix et Rhizoma 8.44 g, Cyperi Rhizoma 8.44 g, Cnidii Rhizoma, Trogopterorum Faeces 5.63 g, Myrrha, Lumbricus 5.63 g, Glycyrrhizae Radix et Rhizoma 2.82 g
**95**	Onkyung-tang	Paeoniae Radix 50 g, Salviae Miltiorrhizae Radix 20 g, Corydalis Tuber 20 g, Angelicae Gigantis Radix 20 g, Evodiae Fructus 15 g, Liriopis seu Ophiopogonis Tuber 15 g, Cnidii Rhizoma 15 g, Cinnamomi Cortex 10 g, Moutan Radicis Cortex 10 g, Pinelliae Tuber 10 g, Glycyrrhizae Radix et Rhizoma 10 g
**96**	Igmosamul-tang	Leonuri Herba 18.75 g, Cyperi Rhizoma 7.5 g, Angelicae Gigantis Radix 5.63 g, Rehmanniae Radix Preparata 5.63 g, Paeoniae Radix 5.63 g, Atractylodis Rhizoma Alba 5.63 g, Achyranthis Radix 5.63 g, Cnidii Rhizoma 3.75 g, Persicae Semen 3.75 g, Cinnamomi Ramulus 3.75 g, Carthami Flos 1.88 g
**97**	Jokyung-san	Leonuri Herba 18.75 g, Cyperi Rhizoma 7.5 g, Angelicae Gigantis Radix 5.63 g, Rehmanniae Radix Preparata 5.63 g, Paeoniae Radix 5.63 g, Atractylodis Rhizoma Alba 5.63 g, Achyranthis Radix 5.63 g, Cnidii Rhizoma 3.75 g, Persicae Semen 3.75 g, Cinnamomi Ramulus 3.75 g, Carthami Flos 1.88 g
**98**	Cheunggyeongsamul-tang	Angelicae Gigantis Radix 5.63 g, Rehmanniae Radix 3.75 g, Scutellariae Radix 3.75 g, Cyperi Rhizoma 3.75 g, Paeoniae Radix 3 g, Coptidis Rhizoma 3 g, Cnidii Rhizoma 1.88 g, Asini Corii Colla 1.88 g, Phellodendri Cortex 1.88 g, Artemisiae Argyi Folium 1.13 g, Glycyrrhizae Radix et Rhizoma 1.13 g
**99**	Cheongyeoljohyeol-tang	Angelicae Gigantis Radix 2.63 g, Cnidii Rhizoma 2.63 g, Paeoniae Radix 2.63 g, Rehmanniae Radix 2.63 g, Coptidis Rhizoma 2.63 g, Cyperi Rhizoma 2.63 g, Persicae Semen 2.63 g, Carthami Flos 2.63 g, Curcumae Rhizoma 2.63 g, Corydalis Tuber 2.63 g, Moutan Radicis Cortex 2.63 g
**100**	Chiljehyangbu-hwan	Cyperi Rhizoma 22.5 g, Angelicae Gigantis Radix 3.75 g, Curcumae Rhizoma 3.75 g, Moutan Radicis Cortex 3.75 g, Artemisiae Argyi Folium 3.75 g, Linderae Radix 3.75 g, Cnidii Rhizoma 1.88 g, Corydalis Tuber 1.88 g, Sparganii Rhizoma 1.88 g, Bupleuri Radix 1.88 g, Carthami Flos 1.88 g, Mume Fructus 1.88 g
**101**	Tonggyungsamul-tang	Angelicae Gigantis Radix 5.63 g, Rehmanniae Radix Preparata 3.75 g, Paeoniae Radix 3.75 g, Cyperi Rhizoma 3.75 g, Curcumae Rhizoma 3.75 g, Sappan Lignum 3.75 g, Akebiae Caulis 3 g, Cnidii Rhizoma 1.88 g, Cinnamomi Cortex 1.88 g, Glycyrrhizae Radix et Rhizoma 1.88 g, Carthami Flos 1.13 g, Persicae Semen 1.13 g
**102**	Tonggyung-tang	Paeoniae Radix 30 g, Typhae Pollen 15 g, Trogopterorum Faeces 10 g, Eupolyphaga 10 g, Corydalis Tuber 10 g, Cyperi Rhizoma 10 g, Meliae Fructus 10 g, Linderae Radix 10 g, Angelicae Gigantis Radix 10 g, Myrrha 3 g, Angelicae Dahuricae Radix 3 g, Foeniculi Fructus 3 g, Glycyrrhizae Radix et Rhizoma 6 g, Evodiae Fructus 3 g, Asiasari Radix et Rhizoma 3 g
**103**	Hyunbooleekyung-tang	Cyperi Rhizoma 11.25 g, Atractylodis Rhizoma, Linderae Radix 5.63 g, Corydalis Tuber 3.75 g, Citri Unshius Pericarpium 3.75 g, Angelicae Gigantis Radix 3.75 g, Paeoniae Radix 3.75 g, Cnidii Rhizoma 3.75 g, Aurantii Fructus Immaturus 3.75 g, Curcumae Rhizoma 3.75 g, Persicae Semen 3.75 g, Cinnamomi Cortex 2.63 g, Aucklandiae Radix 2.63 g, Carthami Flos 2.63 g, Zingiberis Rhizoma Recens 1.5 g
**104**	Hyulbuchuko-tang	Paeoniae Radix 20 g, Rehmanniae Radix Recens 15 g, Paeoniae Radix 15 g, Achyranthis Radix 15 g, Salviae Miltiorrhizae Radix 15 g, Poria Sclerotium 15 g, Atractylodis Rhizoma Alba 15 g, Persicae Semen 12 g, Angelicae Gigantis Radix 12 g, Cnidii Rhizoma 10 g, Platycodonis Radix 10 g, Bupleuri Radix 10 g, Aurantii Fructus Immaturus 10 g, Glycyrrhizae Radix et Rhizoma 10 g, Carthami Flos 8 g

Table S2. Summary of literature search

Searched databases: MEDLINE (via PubMed), and the Cochrane Central Register of Controlled Trials (CENTRAL)
Oriental Medicine Advanced Searching Integrated System (OASIS), and Science-On

Searching date: November 15, 2020

Search strategies
Medline (via PubMed)
➔ (Traditional Korean Medicine OR Traditional Chinese Medicine OR Herbal Medicine) AND Dysmenorrhea AND (systematic review [Filter])
➔ (Traditional Korean Medicine[MeSH] OR Traditional Chinese Medicine[MeSH] OR Herbal Medicine [MeSH]) AND Dysmenorrhea[MeSH] AND (systematic review [Filter])
CENTRAL
➔ "Traditional Chinese medicine"
OR "herbal medicine"
AND "Dysmenorrhea"
➔ #1. MeSH descriptor: [Medicine, East Asian Traditional] explodes all trees.
#2. MeSH descriptor: [dysmenorrhea] explodes all trees.
3. #1 AND #2
OASIS
➔ 월경통 (Korean)
Science-On
➔ 한약 AND 월경통 (Korean)

Inclusion criteria:
Participants: patients with primary dysmenorrhea (over 3 months from onset)
Intervention: herbal medicine (Traditional Korean Medicine or Traditional Chinese Medicine)
Comparison: conventional treatments
Outcomes: Effectiveness (symptoms, quality of life, physical test), safety (laboratory test, physical test)
Study design: Systematic reviews and meta-analysis

Results
Thirteen studies were included in this review. In this study, we reviewed all the included SR and meta-analyses (Figure S1). Table S1 outlines the characteristics of the original SR and meta-analysis.

Table S3. Characteristics of original SR and meta-analysis on primary dysmenorrhea

First author (year)/country	Language	No. of included RCTs	No. of analyzed participants	Intervention	Comparator	Outcomes
Xu, 2020<br>China	English	9	647(333/314)	cinnamon/fennel/ginger	Placebo(8), Mefenamic acid cap(1)	Pain intensity<br>Pain duration
Seo, 2020<br>South Korea	English	13	1214(607/607)	DJS/Modified DJS<br>DJS with DFS(Diclofenac sodium)<br>DJS with moxa	Indomethacin(2), Ibuprofen capsule(3), placebo(1), other HM(6)	Total effective rate, Change of pain, associated symptoms and depression, Consumption of DCF
Li, 2020<br>China	English	39	3982	Siwutang (SWT), THSWT(Taohong SWT), XFSWT(Xiangfu SWT).	Conventional medicine (ibuprofen, indomethacin, diclofenac, norethisterone, and Vitamin E.)	1st: Pain by VAS, VRS, NRS, MPQ(McGill Pain Questionnaire) or BPQ(Brief Pain Questionnaire)<br>2nd: symptom relief by the COX Menstrual Symptom Scale (CMSS), QoL by SF-36, SF-12 or EQ-5L-5D, Response rate, AEs
Lee, 2020<br>South Korea	English	12	970(502/468)	Fennel	Mefenamic acid(6), ibuprofen(1),placebo(5)	Pain by VAS or dichotomous outcomes
Ji, 2020<br>South Korea	English	5	584(275/309)	TST(Taohong Siwu Tang) or modified TST	NSAIDs(Ibuprofen, Indomethacin, and Flufenamic acid), OCs (Estrogen, progesterone)	Response rate*, Recurrence rate, AEs
Leem, 2019<br>South Korea	English	8	1048(524/524)	XZD(Hyeolbuchukeo-tang or Xuefu Zhuyu decoction)	WM(Fenbid, Oryzanol, Thiamine, Indometacin, Naproxen, Diclofenac Sodium)	1st: Response rate*, Pain by VAS<br>2nd: QoL, AEs, Recurrence rate, Symptom score.
Gao, 2017<br>China	English	18	1736(891/845)	Wenjing decoction	NSAIDs: ibuprofen(7), fenbid(5), indomethacin(4), paracetamol & codeine(1), loxoprofen sodium(1)	CER(clinical effective rate), PSD(pain scale for dysmenorrhea), VAS, adverse events
Lee, 2016<br>South Korea	English	4	435	DSS(Danggui Shaoyao San) or modified DSS	WM(NSAIDs(diclopenac, ibuprofen, fenbide) or placebo	Pain by VAS, Response rate, Consumption of DCF, AEs
Lee, 2016<br>South Korea	English	9	893	SFZY(Shaofu Zhuyu decoction) and modified SFZY	WM(ibuprofen, aminopyrine phenacetin, indomethacin)	1st: Pain by VAS, other validated scales, or as a dichotomous outcome. Response rate***<br>2nd: AEs
Zhu, 2008<br>China	English	39	3475	Any CHM, self-designed CHF	WM, placebo, no Tx,, other HM, Atx, massage	1st: pain by VAS, other validated scales, or dichotomous outcomes. Overall reduction in symptoms, AEs.<br>2nd: Laboratory tests (liver and renal function test, hormonal levels), Use of additional medication, Satisfaction of treatment as reported by patients, QoL

*The treatment responses were divided into four categories: “cured”(痊愈), “markedly improved”(显效), “improved”(有效), and “no effect”(无效). The cured, markedly improved, and improved groups were defined as the responder groups. **Add-on composition of formula, + Of the 20 selected studies, only 5 studies were RCTs. *** the response rate; an overall reduction in symptoms(other menstruation-related symptoms) that occurs only during the intervention or occurred as a result of the intervention was measured by changes in dysmenorrhea symptoms and treatment effectiveness and was self-reported, observed or reported by other similar measure

References

Gao, L., Jia, C., Zhang, H., Ma, C. (2017) Wenjing decoction (herbal medicine) for the treatment of primary dysmenorrhea: a systematic review and meta-analysis. Arch. Gynecol. Obstet. 296(4):679-689.

Ji, H. R., Park, K.S., Woo, H.L., Lee, M.J., Yoon, J.G., Lee, H.J., Hwang, D.S., Lee, C.H., Jang, J.B., Lee, J.M. (2020) Herbal medicine (Taohong Siwu Tang) for the treatment of primary dysmenorrhea: A systematic review and meta-analysis. Explore. 16(5):297-303.

Lee, H.W., Ang, L., Lee, M.S., Alimoradi, Z., Kim, E. (2020) Fennel for Reducing Pain in Primary Dysmenorrhea: A Systematic Review and Meta-Analysis of Randomized Controlled Trials. Nutrients. 12(11):3438.

Leem, J., Jo, J., Kwon, C.Y., Lee, H., Park, K.S., Lee, J.M. (2020) Herbal medicine (Hyeolbuchukeo-tang or Xuefu Zhuyu decoction) for treating primary dysmenorrhea: A systematic review and meta-analysis of randomized controlled trials. Medicine. 98(5):e14170.

Li, G., Liu, A., Lin, M., Liao, S., Wen, Z. (2020) Chinese herbal formula siwutang for treating primary dysmenorrhea: A systematic review and meta-analysis of randomized controlled trials. Maturitas. 138:26-35. doi: 10.1016/j.maturitas.2020.03.009. PMID: 32631585.

Seo, J., Lee, H., Lee, D., Jo, H.G. (2020) Dangguijagyag-san for primary dysmenorrhea: A PRISMA-compliant systematic review and meta-analysis of randomized-controlled trials. Medicine. 99(42):e22761.

Xu, Y., Yang, Q., Wang, X (2020). Efficacy of herbal medicine (cinnamon/fennel/ginger) for primary dysmenorrhea: a systematic review and meta-analysis of randomized controlled trials. J. Int. Med. Res. 8(6):300060520936179

Table S4. The reason why we did not include the western medical doctors in the process of developing COS.

As we mentioned in the manuscript, structural conflicts inevitably arise among physicians. Conflict over reimbursement HM has increased, and Western Medical Doctors (WMDs) have opposed the use and reimbursement of HMs in primary care. WMDs have negative biases and conflicts of interest regarding HMs. In addition, political tensions between Korean medical doctors (KMDs) and WMDs are high, and WMD Associations do not allow their members to participate in training and consultation with KMDs. This means that we cannot involve WMDs at any stage. On the other hand, KMDs are trained in both conventional and Korean medicine represented by HMs and acupuncture. In view of this, the author has asked the Society of Korean Medicine, Obstetrics, and Gynecology to recommend stakeholders, including researchers who will use COS and medical professionals who have experience with patients with this condition.

Figure S1. Flow chart of selection process. SR: systematic review

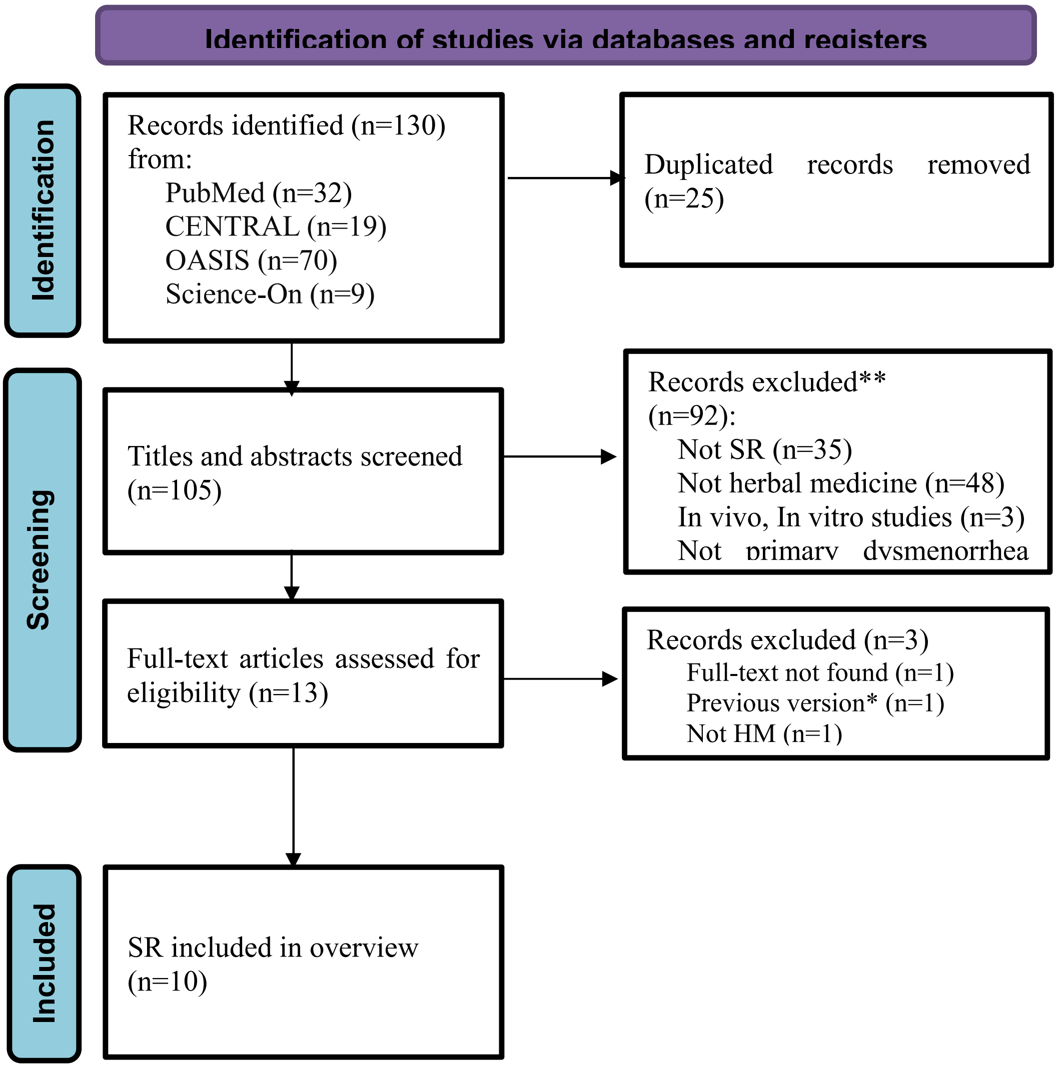

Supplement: Supplementary file 1 [file ijerph-19-15321-s001.zip › ijerph-1925232-supplementary.pdf]
